# Prevalence of Hepatitis C Virus Subgenotypes 1a and 1b in Japanese Patients: Ultra-Deep Sequencing Analysis of HCV NS5B Genotype-Specific Region

**DOI:** 10.1371/journal.pone.0073615

**Published:** 2013-09-17

**Authors:** Shuang Wu, Tatsuo Kanda, Shingo Nakamoto, Xia Jiang, Tatsuo Miyamura, Sueli M. Nakatani, Suzane Kioko Ono, Azusa Takahashi-Nakaguchi, Tohru Gonoi, Osamu Yokosuka

**Affiliations:** 1 Department of Gastroenterology and Nephrology, Chiba University, Graduate School of Medicine, Chiba, Japan; 2 Department of Molecular Virology, Chiba University, Graduate School of Medicine, Chiba, Japan; 3 Department of Gastroenterology, School of Medicine, University of Sao Paulo (USP), São Paulo, Brazil; 4 Medical Mycology Research Center, Chiba University, Chiba, Japan; Saint Louis University, United States of America

## Abstract

**Background:**

Hepatitis C virus (HCV) subgenotypes 1a and 1b have different impacts on the treatment response to peginterferon plus ribavirin with direct-acting antivirals (DAAs) against patients infected with HCV genotype 1, as the emergence rates of resistance mutations are different between these two subgenotypes. In Japan, almost all of HCV genotype 1 belongs to subgenotype 1b.

**Methods and Findings:**

To determine HCV subgenotype 1a or 1b in Japanese patients infected with HCV genotype 1, real-time PCR-based method and Sanger method were used for the HCV NS5B region. HCV subgenotypes were determined in 90% by real-time PCR-based method. We also analyzed the specific probe regions for HCV subgenotypes 1a and 1b using ultra-deep sequencing, and uncovered mutations that could not be revealed using direct-sequencing by Sanger method. We estimated the prevalence of HCV subgenotype 1a as 1.2-2.5% of HCV genotype 1 patients in Japan.

**Conclusions:**

Although real-time PCR-based HCV subgenotyping method seems fair for differentiating HCV subgenotypes 1a and 1b, it may not be sufficient for clinical practice. Ultra-deep sequencing is useful for revealing the resistant strain(s) of HCV before DAA treatment as well as mixed infection with different genotypes or subgenotypes of HCV.

## Introduction

Hepatitis C virus (HCV) infection causes acute and chronic hepatitis, cirrhosis and hepatocellular carcinoma [1,2]. Worldwide, approximately 170 million people are chronically infected with HCV [3]. HCV is a single-stranded RNA virus ~9600 nt in size, and it belongs to the *Flaviviridae family*. HCV genomes are translated into a single open reading frame of ~3000 amino acids, and by cellular and viral encoded-protease are processed into structural (core, E1, E2 and p7) and non-structural proteins (NS2, NS3, NS4A, NS4B, NS5A and NS5B) [4]. HCV is classified into six major genotypes and there are nucleotide differences between HCV genotypes at more than 30%. HCV subgenotypes such as 1a and 1b in HCV genotype 1 also exist, and they are typically different from each other at 20% to 25% of nucleotides [5].

Standard of care (SOC) for chronic hepatitis C is based on a combination of pegylated-interferon and ribavirin for 48 weeks, which leads to only ~50% sustained virological response (SVR) in patients with HCV genotype 1. Contrary to HCV genotype 1, SOC for 24 weeks leads to 70~80% SVR in HCV genotype 2- or 3-infected individuals [4]. Thus, there are differences in treatment response among HCV genotypes.

Recently, two direct-acting antivirals (DAAs) against HCV, telaprevir and boceprevir, were introduced, and they are now available in combination with peginterferon plus ribavirin for treatment of chronic hepatitis C [6]. The addition of these protease inhibitors to SOC leads to significantly higher SVR rates than SOC only in previous-treatment relapsers and untreated patients infected with HCV genotype 1 [6]. It is well known that telaprevir- and boceprevir-based therapies are relatively more effective in HCV subgenotype 1b patients than in those with HCV subgenotype 1a [7,8]. Although we expect to be using other DAAs and interferon-free regimens for therapies of chronic hepatitis C in the near future, the treatment response rate might be low in HCV subgenotype 1a compared to HCV subgenotype 1b infection [9,10]. Thus, HCV genotype or subgenotype is one of the predictors of the response to anti-viral therapy.

In Japan, the proportions of HCV genotypes 1 and 2 are 70% and 30%, respectively. The prevalence of HCV subgenotype 1a in HCV genotype 1, after excluding patients with hemophilia, was reported to be ~1% [11]. However, it is possible that non-responder patients might be associated with HCV subgenotype 1a in Japan. Therefore, it seems important to distinguish between HCV subgenotypes 1a and 1b in Japan as well as in other countries.

To reveal the current prevalence of HCV subgenotype 1a in Japanese HCV genotype 1-infected individuals, we examined HCV subgenotypes 1a and 1b. We also examined the method for identifying HCV genotypes by real-time PCR-based method in the HCV NS5B region [12] and analyzed the specific probe region for HCV subgenotypes 1a and 1b using ultra-deep sequencing.

## Results

### Subgenotyping by one-step real-time PCR with MGB probe in the HCV NS5B region

In the present study, HCV genotypes of all samples were determined by the antibody serotyping method of Tukiyama-Kohara et al. [13,14]. According to this assay, HCV serotypes 1 and 2 correspond to HCV genotypes 1a/1b and 2a/2b [5]. The clinical background of 80 patients was shown in Table 1. All but 2 patients had high viral loads. First, all 80 patients were Japanese, and their HCV subgenotypes were determined by real-time PCR-based method with MGB probe in the HCV NS5B region [12]. HCV subgenotypes were determined in 74 of the 80 patients: 4 (5.0%) and 70 (87.5%) were classified into HCV subgenotypes 1a and 1b, respectively. HCV subgenotypes remained undetermined by this method in the other 6 patients (7.5%).

**Table 1:** Clinical characteristics of HCV genotype 1-infected in the present study.

Number of patients (male/female)	80 (39/41)
Age (years)	51+14
HCV RNA levels (low/high)	2/78
ALT (IU/L)	67+41
WBC (x10^3^/µL)	5.5+1.7
Hemoglobin (g/dL)	14+1.1
Platelet counts (x10^4^/µL)	18+5.8
γ-GTP (IU/L)	53+70
IL28B rs8099917, TT/TG/GG	49/30/1

Note: HCV RNA levels, low: less than 5 log IU/mL; HCV RNA levels, high: equal to and more than 5 log IU/mL; ALT, alanine aminotransferase; WBC, white blood cell count.

### Comparison of the result from HCV subgenotyping by real-time PCR-based method in the HCV NS5B region and that from direct-sequencing by Sanger method in Japanese patients infected with HCV genotype 1

It has been reported that the prevalence of HCV subgenotype 1a was ~1% in Japan [11]. Our result from the real-time PCR-based subgenotyping method suggested that this prevalence might be a little higher although our study population of 80 patients was too small to be considered representative. We tried to perform direct-sequencing by the Sanger method in 10 patients: 4 with HCV subgenotype 1a and 6 with undetermined HCV subgenotypes by real-time PCR-based method [12]. Because the PCR product in 1 of the 4 HCV subgenotype 1a patients could not be amplified, this patient was excluded (Table 2, patient No. 69).

**Table 2:** Results of HCV subgenotype determining by real-time PCR-based method and direct-sequencing by Sanger method.

**Patient No.**	**Real-time PCR-based subgenotyping method**	**Direct-sequencing by Sanger method**
4	undetermined	1b
25	undetermined	1b
37	undetermined	1b
46	1a	1b
47	undetermined	1b
48	1a	1b
66	undetermined	1b
69*	1a	undetermined*
74	1a	1a
78	undetermined	1b

Note: * The PCR product of patient No. 69 could not be obtained before performing direct-sequencing.

TaqMan MGB probes exhibit great differences in Tm values between matched and mismatched probes, providing more accurate allelic discrimination and making for a more sensitive real-time assay [12]. Thus, we then performed direct-sequencing by the Sanger method in 9 patients. As shown in Table 2, only 1 of three patients classified into HCV subgenotype 1a by real-time PCR-based method [12] was confirmed to be HCV subgenotype 1a (Table 2, patient No. 74). All other samples with subgenotype 1a or undetermined by real-time PCR-based method, were confirmed to be HCV subgenotype 1b by direct-sequencing (Table 2). In accordance with the alignments of the MGB probe segments from each patient in comparison to those in H77 and Con1, we found that patients No. 46 and 48 showed an A to G mutation at nucleotide site 8917 and 8918 (reference to Con1), respectively (Table 3).

**Table 3:** Nucleotide sequences in MGB probe segments of each patient.

Refs.	H77 from 8913 to 8926 CAGCTTGAACAGGC	Con1 from 8910 to 8923 CAACTTGAAAAAGC
No. 4	---------A----	--G--------G--
No. 25	--A------A-A--	--------------
No. 37	--A------A-A--	--------------
No. 46	-------G-A-A--	--G----G------
No. 47	--A------A-A--	--------------
No. 48	--A-----GA-A--	--------G-----
No. 66	--A------A-A--	--------------
No. 74	--------------	--G------C-G--
No. 78	--A------A-A--	--------------

Reference sequences (Refs) were obtained from HCV subgenotype 1a (H77) and HCV subgenotype 1b (Con1). GenBank accession No.: H77, AF009606.1; Con1, AJ238799.1.

#### Phylogenetic tree analysis

**Figure 1:**
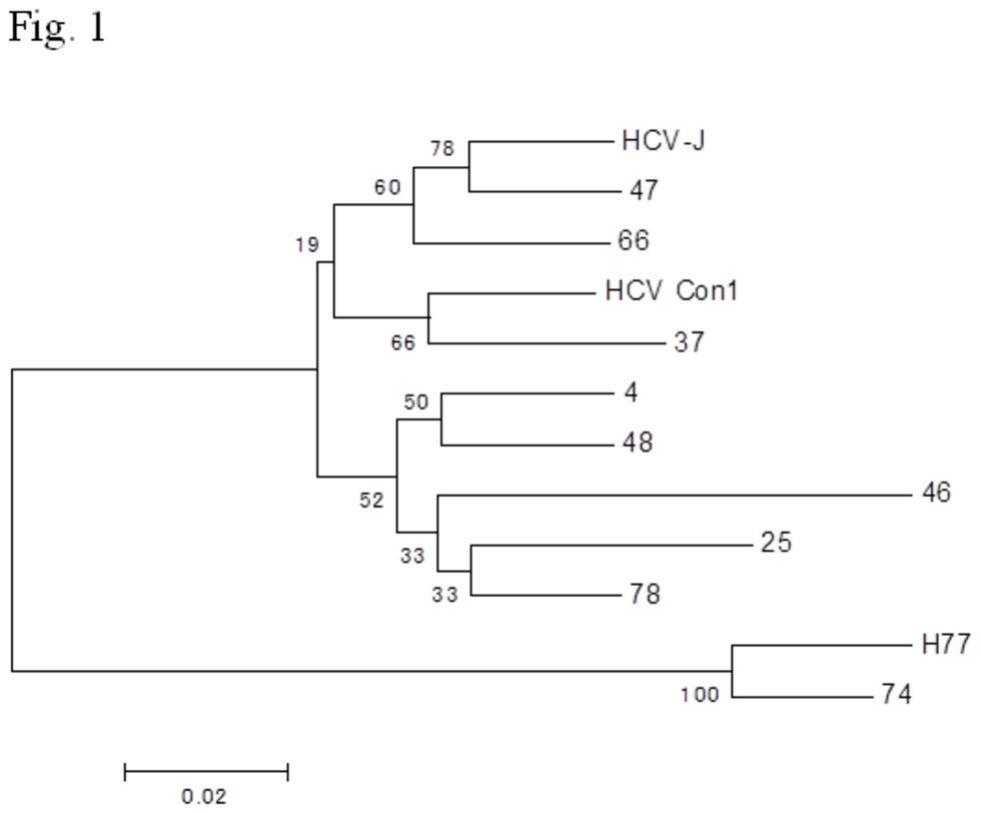
Phylogenetic tree analysis by the Neighbor-Joining (NJ) method. The numbers at the branches are confidence values based on Felsenstein’s bootstrap analysis (500 replicates) with MEGA version 4 [28]. HCV HCV-J, HCV Con1 and HCV H77 strains belong to HCV subgenotypes 1b, 1b and 1a, respectively. GenBank accession No: HCV-J, D90208.1; Con1, AJ238799.1; H77, AF009606.1.

We also constructed a phylogenetic tree based on the 9 patients’ sequences of about 424 bp length containing the HCV NS5B probe region obtained using direct-sequencing by the Sanger method. The phylogenetic tree showed that only one patient, No. 74, belonged to HCV subgenotype 1a, while the others all clustered to HCV subgenotype 1b (Figure 1).

#### Calculation of PCR and Roche/454 GS Junior sequencer error rates

In order to ensure that errors introduced by PCR as well as errors inherent to the Roche/454 pyrosequencing technology were below our minimum variant frequency threshold of 1%, we sequenced the PCR products from 10^3^ to 10^4^ copies of control plasmid and found no mutations, indicating similar error rates lower than 1%.

Sequencing read lengths of No. 4, 25, 37, 46, 47, 48, 64, 74 and 78 were 5151, 3725, 6075, 5145, 5071, 3939, 8990, 4002 and 9227, respectively. The coverage numbers were 2078-9195 at each position.

#### Performance comparison of ultra-deep sequencing and Sanger direct-sequencing

To explore the difference between ultra-deep sequencing and direct-sequencing by the Sanger method, we compared the differences in nucleotide sequences between the two methods (Tables 3 4). Direct-sequencing showed that only 3 of 8 HCV subgenotype 1b isolates (37.5%) had nucleotide sequence variations, compared to MGB probe sequence, according to HCV Con1. Concerning HCV subgenotype 1a isolate, direct-sequencing showed no nucleotide sequence variations, compared to MGB probe sequence, according to HCV H77.

**Table 4:** Analysis of nucleotide sequence variations (%) in MGB probe segments by ultra-deep sequencing.

Refs.	Variants	Max	No. 4	No. 25	No. 37	No. 46	No. 47	No. 48	No.66	No. 74	No. 78
Con1	8912:A/G	100	***99.28***	0	***27.01***	***100***	0	0	0	NA	0.07
Con1	8913.5:-/G	30	0	0.05	0	0	***30***	0	0	NA	0
Con1	8915.5:T/C	0.52	0.52	0	0	0	0	0	0	NA	0
Con1	8915.5:-/A	70	0	0.83	0.26	0.29	***70***	0	0	NA	0
Con1	8917:A/G	99.84	0	0	0	***99.84***	***30***	0	0	NA	0.03
Con1	8918:A/G	99.92	0	0	0	0	0	***99.92***	0	NA	0
Con1	8919:A/G	0.52	0.52	0	0	0	0	0	0	NA	0
Con1	8920:A/G	2.33	***2.33***	0	0	0	0	0	0	NA	0
Con1	8921:A/G	85.79	***85.79***	0	0	0	0	0	0	NA	0
Con1	8921.5:-/G	0.44	0	0.4	0.07	0	***70***	0.08	0	NA	0.44
Con1	8922:G/A	1.11	***1.11***	0	0	0	0	0	0	NA	0
Con1	8922.5:-/G	1.09	0.97	0	0	0	0	0	0	NA	***1.09***
H77	8915:G/A	1.9	NA	NA	NA	NA	NA	NA	NA	***1.9***	NA

Reference sequences (Refs) were obtained from HCV subgenotype 1a (H77) and HCV subgenotype 1b (Con1). GenBank accession No.: H77, AF009606.1; Con1, AJ238799.1. Bold oblique indicates significant change (1%) from reference sequences. Number indicates the nucleotide changes that were identical in direct-sequencing by Sanger Method.

However, analysis of ultra-deep sequences showed that 6 of 8 HCV subgenotype 1b isolates (75%) had nucleotide sequence variations (1%), compared to MGB probe sequence, according to HCV Con1. In No. 37 and No. 47, respectively, one mutation [8912:A/G mutation (27%)] and four mutations [8913.5:/G (30%), 8915.5:/A (70%), 8917:A/G (30%) and 8921.5:/G (70%)] were identified in ultra-deep sequences. Similarly, in No. 78, one mutation [8922.5:/G (1.09%)] was identified (Table 4).

Even in the HCV subgenotype 1a isolate, analysis of ultra-deep sequences showed one nucleotide sequence variation (1%), compared to MGB probe sequence, according to HCV H77 (Table 4). We also compared nucleotide sequence variations between MGB probe and non-MGB probe regions (data not shown). In 8 HCV subgenotype 1 patients, we found no difference in nucleotide sequence variations per site between MGB probe and non-MGB probe regions (0.16 + 0.14 vs. 0.074 + 0.029, *P = 0.11*).

## Discussion

In the present study, we used a novel HCV subgenotyping method based on real-time PCR [12] in Japanese patients infected with HCV genotype 1. Real-time PCR-based HCV subgenotyping method seems fair for differentiating the HCV subgenotypes 1a and 1b. But it might not be good enough for clinical practice, as HCV subgenotypes were only determined in 74 of 80 patients (92.5%), despite our study population being small. As direct-sequencing could only apply to cases in which the PCR products were obtained by RT-PCR methods, real-time PCR-based HCV subgenotyping method might be useful if the PCR products cannot be amplified.

Ultra-deep pyrosequencing is a promising technology for characterizing and detecting minor variants and drug resistant variants [15]. Pyrosequencing could be useful for cost-effective estimation of drugs for treatment and disease controls [16]. We also performed analysis of ultra-deep sequences in the probe region of HCV NS5B. In comparison with direct-sequencing by the Sanger method, we found other changes of nucleotide sequences in this region. It is possible that these variations might have some effects on the results by the real-time PCR-based HCV subgenotyping method. Our results support the previous studies showing that ultra-deep sequencing technologies are powerful tools for obtaining more profound insight into chronic HCV infection [17-19]. Our study also showed that analysis of ultra-deep sequences provided additional information that we could not obtain using direct-sequencing by the Sanger method. We also found minor sequence variations (1%), although we do not know whether these sequences are significant or what roles they play at this time. Further studies will be needed.

We estimated the prevalence of HCV subgenotype 1a at 1.2-2.5% of HCV genotype 1 patients in Japan. It is well known that some patients treated with telaprevir experienced viral breakthrough with telaprevir-resistant strains, most of which harbored the R155T/K mutations. Only a single nucleotide change is needed to produce the amino acid change at codon 155 in HCV subgenotype 1a, while two nucleotide changes are required in HCV subgenotype 1b. This might be one of the reasons why resistance mutation was selected more frequently in HCV subgenotype 1a than in HCV subgenotype 1b in the case of telaprevir use [20], although in the ELECTRON study of NS5B inhibitor sofosbuvir, no differential resistance was observed between genotypes 1a and 1b despite 89% of the subjects being the HCV genotype 1a population [21]. Of interest, it has been reported that there are different impacts of IL28B variants on the treatment response to SOC between HCV subgenotype 1a and 1b strains together with HIV co-infection [22]. It will be important to determine HCV subgenotype 1a or 1b before commencing treatment for HCV genotype 1.

It was reported that Versant HCV genotype assay (LIPA version.1 probe assay based on 5’ UTR, Siemens, Tarrytown, NY, USA) could not determine HCV subgenotypes in 23% of HCV-infected patients in Brazil [12]. Thomas et al. reported that upgrading LIPA to the newer version 2.0 assay resulted in an increase in identification of genotype 1a by 18.5% [23]. Improved technology of HCV genotyping and subgenotyping could lead to accurate HCV genotype identification and HCV subtyping. Ultra-deep sequencing is useful for revealing the mutations in viral genome of HCV-infected individuals. However, it might not be useful as a method for differentiating HCV subgenotype 1a and 1b, although their combination as well as next-generation sequencing [24] could perhaps lead to better results in the determination of HCV subgenotypes. It could be used to find the resistant strain(s) of HCV before DAA treatment and mixed infection with different genotypes or subgenotypes of HCV, although Akuta et al. [25] reported that it was difficult to predict at baseline the emergence of telaprevir-resistant variants after commencement of therapy in prior non-responders of HCV genotype 1, even with the use of ultra-deep sequencing.

It was reported that phylogenetic analysis revealed different clusters between the HCV subgenotype 1b strains obtained in Japan and those in Brazil [26]. In fact, the distribution of HCV genotypes differs in various countries [27]. The discrepancy between the results of the present and previous studies [12] might be due to the differences between HCV subgenotype 1b strains in Japan and in Brazil.

In conclusion, the method for identifying HCV subgenotype 1a or 1b by the real-time PCR-based method in the HCV NS5B region was examined in Japanese patients infected with HCV genotype 1, and we estimated a 1.2-2.5% prevalence of HCV subgenotype 1a among HCV genotype 1 patients in Japan. We also analyzed the specific probe regions for HCV subgenotypes 1a and 1b, of which we could not determine HCV subgenotypes using specific probes, and uncovered the mutations that direct-sequencing by the Sanger method could not reveal, existing in their probe regions using ultra-deep sequencing. Ultra-deep sequencing is useful for revealing the resistant strain(s) of HCV before DAA treatment as well as mixed infection with different genotypes or subgenotypes of HCV.

## Materials and Methods

### Patients

Sera from 80 consecutive treatment-naïve chronic hepatitis C genotype 1 patients with well-characterized clinical follow-up were selected from a chronic hepatitis C database at Chiba University Hospital, Chiba, Japan. All patients were negative for hepatitis B and HIV antibodies. Sera from patients were stored at -20°C until analysis. This study was approved by the Ethics Committee, Chiba University, Graduate School of Medicine, Chiba, Japan (permission numbers 1462 and 282), and conformed to the Helsinki Declaration. Written informed consent was obtained from all patients before enrollment in this study.

### RNA extraction

Total RNA was extracted from 140 µL of each serum sample using a QIAamp Viral RNA Mini Kit (Qiagen, Tokyo, Japan) according to the manufacturer’s instructions. RNA was eluted in 60 µL of elution buffer and quantified using a NanoDrop Lite spectrophotometer (Thermo Scientific, Madison, WI, USA).

### HCV subgenotyping by real-time PCR-based methods in the HCV NS5B region

Reactions were performed in a final volume of 50 µL using the Superscript™ III Platinum one-step quantitative RT-PCR system (Invitrogen, Carlsbad, CA, USA) as previously described [12]. The designed primers and MGB probes shown in Table 5 and the real-time PCR HCV subgenotyping protocol were used according to the previous description [12]. The reaction mixture was prepared in two fractions. The first fraction (F1) consisted of 18.5 µL of 2X reaction mix (6 mM MgSO_4_, and 0.4 mM of each dNTP), 1.0 µL of *Taq* mix (Superscript™III RT, Platinum *Taq* Mix, Invitrogen), 200 nM of reverse primer (R56_1), and 0.5 µL of RNase Out (Invitrogen). Twenty-one microliters of F1 was added to optical tubes. The second reaction mix fraction (F2) consisted of 4.4 µL of 2X reaction mix, 200 nM of forward primer (F56_1), 1.5 µL of 50 mM MgSO_4_, 0.1 µL of ROX (25 µM), and 200 nM of each probe (against either genotype 1a or 1b). A final F2 volume of 10 µL was transferred to another optical tube. After the addition of 19 µL of RNA, F1 was carefully added to F2. Reverse transcription was carried out in a TakaRa PCR Thermal Cycler (TaKaRa, Ohtsu, Shiga, Japan) at 55°C for 35 min with the thermocycler cover open. Following this, the tubes were briefly centrifuged and real-time PCR was carried out in a 7300 Real-Time PCR System (Applied Biosystems, Foster, CA, USA) using the following cycling parameters: 50°C for 2 min; 95°C for 10 min; and 40 cycles at 95°C for 15 sec, 50°C for 30 sec, and 60°C for 1 min. The total time required for performing this assay was only 2 hours, as previously described [12].

**Table 5:** Probes and primers used for real-time PCR-based method.

**Name**	**Fluorophore**	**Sequence**	**Quencher**
1a66	6-FAM	5’-CAGCTTGAACAGGC-3’ MGB	NFQ-MGB
1b266	VIC	5’-CAACTTGAAAAAGC-3’ MGB	NFQ-MGB
F56_1	-	5’-CACACTCCAGTYAAYTCCTGG -3’	-
R56_1	-	5’-CWMCTGGAGAGTAACTGTGGAG -3’	-

Probes and primers used for HCV subgenotypes 1a and 1b in HCV subgenotyping assay by real-time PCR-based method in the HCV NS5B region [12]. Probes 1a66 and 1b266 were used for the detection of HCV subgenotypes 1a and 1b, respectively.

### cDNA synthesis and amplification by PCR for ultra-deep sequencing

To perform ultra-deep sequencing of the MGB probe region in HCV NS5B, we used the HPLC-purified specific primers shown in Table 6. cDNA was synthesized with R56_1 (antisense) for 1 cycle at 55°C for 30 min and 85°C for 5 min using a Transcript high-fidelity cDNA synthesis kit (Roche, Tokyo, Japan). Then amplification was performed with Pr1 (sense) and R56_1 (antisense) for 35 cycles at 95°C for 30 sec, 55°C for 30 sec, and 72°C for 60 sec using a FastStart high-fidelity PCR system, dNTPack kit (Roche).

**Table 6:** Probes and primers for sequencing used in the present study.

**Name**	**Sequence**	Location*
Pr1 (sense)	5’-GTATGATACCCGCTGCTTTGA-3’	nt. 8253-8272
Pr2 (sense)	5’-TTCACGGAGGCTATGAC-3’	nt. 8613-8629
Pr2 (antisense)	5’-GTCATAGCCTCCGTGAA-3’	nt. 8629-8613
R56_1 (antisense)	5’-CTGGAGAGTAACTGTGGAG-3’	nt. 9036-9018

Primers used in direct-sequencing by Sanger method and ultra-deep sequencing of HCV NS5B region. * The location of primers corresponding to Con1.

Then, the first PCR product was further amplified with two inner primer sets using the FastStart high-fidelity PCR system, dNTPack kit (Roche). Set one was Pr2 (sense primer) and R56_1 (antisense primer), and the other was Pr1 (sense primer) and Pr2 (antisense primer). Considering the MGB probe locations at nt 8913-8926(1a) in reference sequence HCV subgenotype 1a, strain H77 (AF009606.1), and nt8910-8923(1b) in reference sequence HCV subgenotype 1b, strain Con1 (AJ238799.1), primer set one was used for PCR. The PCR conditions were as follows: 35 cycles at 95°C for 30 sec, 55°C for 30 sec, and 72°C for 60 sec. Amplified products were separated by agarose gel electrophoresis and purified using a high pure PCR clean-up micro kit (Roche). Each amplicon was quantified using a NanoDrop Lite spectrophotometer (Thermo Scientific), and all amplicons from a single viral genome were pooled together at equimolar ratios. Each pool was then quantitated, and approximately 500 ng of each was used in a fragmentation reaction mix using a GS FLX Titanium Rapid Library Preparation Kit (Roche). Final libraries representing each genome were characterized for average size by using an Agilent High Sensitivity DNA kit on Agilent 2100 Bioanalyzer (Agilent Technologies, Loveland, CO, USA). 4 x 10^7^ molecules of these final DNA libraries were then subjected to emulsion PCR, and enriched DNA beads were loaded onto a picotiter plate and pyrosequenced with a Roche/454 GS Junior sequencer using Titanium chemistry (454 Life Sciences Corp., Branfold, CT, USA). GS Amplicon Variant Analizer Version 2.7 (Roche) was used for read mapping and calculating variant frequencies at each nucleotide position according to reference sequence HCV subgenotype 1a, strain H77 or HCV subgenotype 1b, strain Con1.

### Direct-sequencing by Sanger method

Then, we amplified the first PCR product using primer set one and TaKaRa Ex Taq (TaKaRa). The PCR conditions were as follows: 40 cycles at 98°C for 10 sec, 55°C for 30 sec, and 72°C for 60 sec; the last cycle followed at 72°C for 7 min. Sanger sequencing was performed using a BigDye(R) Terminator v3.1 Cycle Sequencing Kit (Life Technologies, Tokyo, Japan). Sequences were detected using Applied Biosystems 3730xl. Nucleotide sequences were analyzed by GENETYX 10 (GENETYX Corp., Tokyo, Japan) and imported to MEGA version 4 [28], which was used to align the sequences, according to reference sequences.

### Construction of phylogenetic trees

A fragment of about 424-bp length containing the HCV NS5B region was used for phylogenetic analysis. Phylogenetic trees were inferred using the Neighbor-Joining (NJ) model and robustness of the tree branches was tested using bootstrap analysis (500 replicates) with MEGA version 4 [28].

### Nucleotide sequence accession number

All sequence reads have been deposited in the DNA Data Bank of Japan (DDBJ) Sequence Read Archive under accession number DRA001077.

### Statistical analysis

Data were expressed as mean + standard deviation. We used univariative analyses, applying Student’s t-test or Chi-square test as appropriate. *P 0.05* was considered statistically significant.

## References

[1] Di Bisceglie AM (1997) Hepatitis C and hepatocellular carcinoma. Hepatology 26 Suppl 1(3: 34S-38S. PubMed: 9305661. 9305661 10.1002/hep.510260706

[2] Saito I, Miyamura T, Ohbayashi A, Harada H, Katayama T et al. (1990) Hepatitis C virus infection is associated with the development of hepatocellular carcinoma. Proc Natl Acad Sci U S A 87: 6547-6549. doi:10.1073/pnas.87.17.6547. PubMed: 2168552. 2168552 10.1073/pnas.87.17.6547 PMC54573

[3] Brownell J, Polyak SJ (2013) Molecular pathways: hepatitis C virus, CXCL10, and the inflammatory road to liver cancer. Clin Cancer Res 19: 1347-1352. doi:10.1158/1078-0432.CCR-12-0928. PubMed: 23322900. 23322900 10.1158/1078-0432.CCR-12-0928 PMC3602344

[4] Kanda T, Imazeki F, Yokosuka O (2010) New antiviral therapies for chronic hepatitis C. Hepatol Int 4: 548-561. doi:10.1007/s12072-010-9193-3. PubMed: 21063477. 21063477 10.1007/s12072-010-9193-3 PMC2940000

[5] Simmonds P, Bukh J, Combet C, Deléage G, Enomoto N et al. (2005) Consensus proposals for a unified system of nomenclature of hepatitis C virus genotypes. Hepatology 42: 962-973. doi:10.1002/hep.20819. PubMed: 16149085. 16149085 10.1002/hep.20819

[6] Kanda T, Yokosuka O, Omata M (2013) Treatment of hepatitis C virus infection in the future. Clin Transl Med 2: 9 PubMed: 23577631. 23577631 10.1186/2001-1326-2-9 PMC3637513

[7] Kieffer TL, De Meyer S, Bartels DJ, Sullivan JC, Zhang EZ et al. (2012) Hepatitis C viral evolution in genotype 1 treatment-naïve and treatment-experienced patients receiving telaprevir-based therapy in clinical trials. PLOS ONE 7: e34372. doi:10.1371/journal.pone.0034372. PubMed: 22511937. 22511937 10.1371/journal.pone.0034372 PMC3325239

[8] Bacon BR, Gordon SC, Lawitz E, Marcellin P, Vierling JM et al. (2011) Boceprevir for previously treated chronic HCV genotype 1 infection. N Engl J Med 364: 1207-1217. doi:10.1056/NEJMoa1009482. PubMed: 21449784. 21449784 10.1056/NEJMoa1009482 PMC3153125

[9] Chayama K, Takahashi S, Toyota J, Karino Y, Ikeda K et al. (2012) Dual therapy with the nonstructural protein 5A inhibitor, daclatasvir, and the nonstructural protein 3 protease inhibitor, asunaprevir, in hepatitis C virus genotype 1b-infected null responders. Hepatology 55: 742-748. doi:10.1002/hep.24724. PubMed: 21987462. 21987462 10.1002/hep.24724

[10] Lok AS, Gardiner DF, Lawitz E, Martorell C, Everson GT et al. (2012) Preliminary study of two antiviral agents for hepatitis C genotype 1. N Engl J Med 366: 216-224. doi:10.1056/NEJMoa1104430. PubMed: 22256805. 22256805 10.1056/NEJMoa1104430

[11] Hayashi K, Katano Y, Kuzuya T, Tachi Y, Honda T et al. (2012) Prevalence of hepatitis C virus genotype 1a in Japan and correlation of mutations in the NS5A and single-nucleotide polymorphism of interleukin-28B with the response to combination therapy with pegylated-interferon-alpha 2b and ribavirin. J Med Virol 84: 438-444. doi:10.1002/jmv.23207. PubMed: 22246829. 22246829 10.1002/jmv.23207

[12] Nakatani SM, Santos CA, Riediger IN, Krieger MA, Duarte CA et al. (2010) Development of hepatitis C virus genotyping by real-time PCR based on the NS5B region. PLOS ONE 5: e10150. doi:10.1371/journal.pone.0010150. PubMed: 20405017. 20405017 10.1371/journal.pone.0010150 PMC2854153

[13] Tsukiyama-Kohara K, Yamaguchi K, Maki N, Ohta Y, Miki K et al. (1993) Antigenicities of Group I and II hepatitis C virus polypeptides—molecular basis of diagnosis. Virology 192: 430-437. doi:10.1006/viro.1993.1058. PubMed: 7678473. 7678473 10.1006/viro.1993.1058

[14] Tanaka T, Tsukiyama-Kohara K, Yamaguchi K, Yagi S, Tanaka S et al. (1994) Significance of specific antibody assay for genotyping of hepatitis C virus. Hepatology 19: 1347-1353. doi:10.1002/hep.1840190605. PubMed: 7514558. 7514558

[15] Wang C, Mitsuya Y, Gharizadeh B, Ronaghi M, Shafer RW (2007) Characterization of mutation spectra with ultra-deep pyrosequencing: application to HIV-1 drug resistance. Genome Res 17: 1195-1201. doi:10.1101/gr.6468307. PubMed: 17600086. 17600086 10.1101/gr.6468307 PMC1933516

[16] Eriksson N, Pachter L, Mitsuya Y, Rhee SY, Wang C et al. (2008) Viral population estimation using pyrosequencing. PLOS Comput Biol 4: e1000074 PubMed: 18437230. 18437230 10.1371/journal.pcbi.1000074 PMC2323617

[17] Nasu A, Marusawa H, Ueda Y, Nishijima N, Takahashi K et al. (2011) Genetic heterogeneity of hepatitis C virus in association with antiviral therapy determined by ultra-deep sequencing. PLOS ONE 6: e24907. doi:10.1371/journal.pone.0024907. PubMed: 21966381. 21966381 10.1371/journal.pone.0024907 PMC3178558

[18] Ninomiya M, Ueno Y, Funayama R, Nagashima T, Nishida Y et al. (2012) Use of illumina sequencing technology to differentiate hepatitis C virus variants. J Clin Microbiol 50: 857-866. doi:10.1128/JCM.05715-11. PubMed: 22205816. 22205816 10.1128/JCM.05715-11 PMC3295113

[19] Thomas XV, de Bruijne J, Sullivan JC, Kieffer TL, Ho CK et al. (2012) Evaluation of persistence of resistant variants with ultra-deep pyrosequencing in chronic hepatitis C patients treated with telaprevir. PLOS ONE 7: e41191. doi:10.1371/journal.pone.0041191. PubMed: 22848441. 22848441 10.1371/journal.pone.0041191 PMC3407168

[20] Soriano V, Vispo E, Poveda E, Labarga P, Martin-Carbonero L (2011) Directly acting antivirals against hepatitis C virus. J Antimicrob Chemother 66: 1673-1686. doi:10.1093/jac/dkr215. PubMed: 21652618. 21652618 10.1093/jac/dkr215

[21] Stedman CA (2013) Current prospects for interferon-free treatment of hepatitis C in 2012. J Gastroenterol Hepatol 28: 38-45. doi:10.1111/jgh.12019. PubMed: 23137126. 10.1111/jgh.12028 23137126

[22] Vispo E, Rallon NI, Labarga P, Barreiro P, Benito JM et al. (2012) Different impact of IL28B polymorphisms on response to peginterferon-alpha plus ribavirin in HIV-positive patients infected with HCV subtypes 1a or 1b. J Clin Virol 55: 58-61. doi:10.1016/j.jcv.2012.05.012. PubMed: 22727259. 22727259 10.1016/j.jcv.2012.05.012

[23] Thomas LB, Foulis PR, Mastorides SM, Djilan YA, Skinner O et al. (2012) Hepatitis C genotype analysis: results in a large veteran population with review of the implications for clinical practice. Ann Clin Lab Sci 42: 355-362. PubMed: 23090730. 23090730

[24] Chevaliez S, Rodriguez C, Pawlotsky JM (2012) New virologic tools for management of chronic hepatitis B and C. Gastroenterology 142: 1303-1313.e1 PubMed: 22537437. 10.1053/j.gastro.2012.02.027 PMC3477068 22537437

[25] Akuta N, Suzuki F, Seko Y, Kawamura Y, Sezaki H et al. (2013) Emergence of telaprevir-resistant variants detected by ultra-deep sequencing after triple therapy in patients infected with HCV genotype 1. J Med Virol 85: 1028-1036. doi:10.1002/jmv.23579. PubMed: 23588728. 23588728 10.1002/jmv.23579

[26] Nakano T, Lu L, Pybus O (2004) Viral gene sequences reveal the variable history of hepatitis C virus infection among countries. J Infect Dis 190: 1098-1108. doi:10.1086/422606. PubMed: 15319860. 15319860 10.1086/422606

[27] Takada N, Takase S, Takada A, Date T (1993) Differences in the hepatitis C virus genotypes in different countries. J Hepatol 17: 277-283. doi:10.1016/S0168-8278(05)80205-3. PubMed: 8391038. 8391038 10.1016/s0168-8278(05)80205-3

[28] Wu S, Imazeki F, Kurbanov F, Fukai K, Arai M et al. (2011) Evolution of hepatitis B genotype C viral quasi-species during hepatitis B e antigen seroconversion. J Hepatol 54: 19-25. doi:10.1016/j.jhep.2010.06.018. PubMed: 20932594. 20932594 10.1016/j.jhep.2010.06.018

